# In Silico Molecular Docking of Phytochemicals of Murraya koenigii Against Streptococcus mutans

**DOI:** 10.7759/cureus.53679

**Published:** 2024-02-05

**Authors:** Kancharla Uma Maheswari, Sathish Sankar

**Affiliations:** 1 Department of Microbiology, Saveetha Dental College and Hospitals, Saveetha Institute of Medical and Technical Sciences (SIMATS) Saveetha University, Chennai, IND

**Keywords:** dental, disease, health, streptococcus mutans, murraya koenigii

## Abstract

Background

The curry leaf tree, *Murraya koenigii*, is a tropical to subtropical tree in the family Rutaceae that is native to Asia. The plant parts are shown to have potential antimicrobial, antioxidant, antifungal, antidiarrheal, antidiabetic, anticancer, and anti-inflammatory properties. *Streptococcus mutans* is a facultative anaerobic, Gram-positive cocci, a common inhabitant of the human oral cavity that forms biofilms, contributing to dental caries.

Aim

The study aimed to analyze the inhibitory potential of phytocompounds in *M. koenigii* against the oral pathogen *S. mutans*.

Materials and methods

The protein and ligand were prepared, and molecular docking was carried out using the Hex protein docking server. The PyMOL program was used to view, analyze, and annotate the docked complex. The interaction of the drug, including the mechanism of action, and predicted adverse effects were predicted using the Way2Drug PASS Online server. The absorption, distribution, metabolism, excretion, and toxicity properties of the drug candidates were analyzed using the SwissADME online server.

Results

The study identified O-methyl murrayamine, koenigine, koenigicine, and murrayone as having inhibitory potential against the glycosyltransferase protein of *S. mutans. *Among the four compounds analyzed for docking, koenigicine had the lowest E-score, indicating a strong interaction with the receptor. Among the four compounds analyzed, murrayone had a high topological polar surface area score, while all four compounds had similar bioavailability scores.

Conclusion

This study concluded that O-methyl murrayamine, koenigine, koenigicine, and murrayone exhibit potent inhibitory potential against *S. mutans*. *M. koenigii*leaf extract can be used in toothpaste as an antibacterial agent to protect teeth against dental caries. These findings are important for the potential use of the above compound to act as an anticariogenic agent in oral health applications.

## Introduction

*Murraya koenigii*, which is commonly referred to as curry leaves, is a tropical to subtropical tree belonging to the family Rutaceae and is native to Asia, especially in India and Sri Lanka [1]. The leaves of the plants are an important ingredient in Indian cuisine and also play a significant role in traditional Indian medicine. Different plant parts, including leaves in either dried form or extracted essential oil, are being used as flavoring agents in bathing soaps and are extensively used in cosmetic aromatherapy [2,3]. *M. koenigii* has been reported to possess antioxidant, antibacterial, antifungal, antidiarrheal, antidiabetic, and anti-inflammatory properties. The leaves of the plant have been used in traditional Indian cooking as one of the most important ingredients in almost all recipes for several centuries. Certain compounds of *M. koenigii* (neplanocin A) are also reported to have potential mosquitocidal effects by disrupting larval growth and are rich in vitamins (C, A, B, and E).

*Streptococcus mutans* is a Gram-positive cocci and facultative anaerobe commonly found in the human oral cavity and is an important and major cause of dental caries [4]. The fall in pH in the mouth creates a favorite environment for the pathogen and causes erosive and progressive tooth decay, a leading cause of tooth loss in all age groups. *S. mutans* is considered the most cariogenic of all oral streptococci and plays a vital role in decalcification [5,6]. In the early stages of the disease, there are bacteria on the dental enamel surface. Bacteria invade hard tissue only after severe demineralization or cavitation. Demineralized areas on the tooth surface, called carious lesions, rather than a disease, are considered a reflection of active bacterial growth and biofilm formation on the dental surface [7]. Bacteria metabolize different types of carbohydrates and therefore create a low pH in the mouth. It is imperative to inhibit the growth of *S. mutans* without much use of antibiotics to prevent tooth decay and maintain oral health.

Specifically, traditional medicinal plant extracts or phytochemicals have been demonstrated to lessen dental plaque formation, impact bacterial adherence to surfaces, and lessen the symptoms of oral illnesses. With *S. mutans* being part of the oral flora and an important oral pathogen causing dental caries, inhibition of such pathogen using medical plant extracts is necessary, which reduces the use of antimicrobials as well. While efficient, approved drugs targeting biofilm production or combating drug-resistant pathogens are currently lacking, the search for natural phytocompounds that effectively counteract biofilm formation is warranted.

In the present study, phytochemicals identified from *M. koenigii *were tested for their ability to inhibit the growth of *S. mutans* through binding and inhibition of the glycosyltransferase protein, an important enzyme involved in the biosynthesis of sugars and glycoconjugates, through catalysis of the activated sugars using a bioinformatic approach.

## Materials and methods

The study was conducted at the Centre for Infectious Diseases at Saveetha Dental College and Hospitals, Saveetha Institute of Medical and Technical Sciences (SIMATS) in Chennai, India. It was carried out using publicly available computational resources for natural phytocompound-based drug discovery. The three-dimensional structures of the glycosyltransferase protein of *S. mutans* and the compounds of *M. koenigii *were used for in silico analysis of protein interaction.

Protein-ligand preparation

Phytochemicals identified from *M. koenigii* (Table 1) were selected for the study. The PubChem database was used to retrieve the chemical structures in structured data format (SDF). These were then converted to Protein Data Bank (PDB) format using the PyMOL Molecular Graphics System software program. The glycosyltransferase protein of *S. mutans* was selected as the protein and obtained from the RCSB Protein Data Bank database. The three-dimensional structure of the protein was downloaded as a PDB file. Using Autodock MGL tools, water molecules were removed, Kollman partial charges and polar hydrogen atoms were added, and the PDBQT file format was prepared for further analysis.

**Table 1:** List of predominant compounds of M. koenigii leaves used in this study

List of compounds of *M. koenigii*	
O-methyl murrayamine	Mahanimboline
Koenigine	Murrayazolinol
Koenigicine	Bismahanine
Murrayone	Isolongifolene
Mahanimbicine	Girinimbilol
Mahanimbine	Bismurrayafoline E
Mahanimbinine	Phebalosin

Molecular docking

The protein and ligand were subjected to molecular docking to predict the binding efficacy of the compounds for their potential inhibitory activity. Docking was performed using the AutoDock software program version 4.2.6 (The Scripps Research Institute, La Jolla, California, USA). Ligands were individually docked against the target glycosyltransferase protein. The active sites were identified using the active site prediction tool [8]. The server computes the cavities in a given protein. The protein was fed as input, and the resulting active sites were identified. In this analysis, a grid of 60 × 60 × 60 with 0.5 Å spacing was set with the Lamarckian genetic algorithm. The grid parameters were set with default settings. Docking scores for the best 10 models were taken into consideration and analyzed based on the score, binding efficacy, and the number of hydrogen bonds. The best interaction with the lowest energy with the maximum number of interactions was selected for further analysis. The protein interaction with the ligand was analyzed using the PyMOL software program.

Drug-likeliness and absorption, distribution, metabolism, excretion, and toxicity (ADMET) properties

The compounds found to have high binding interactions with the protein were selected for the prediction of drug likeliness and their ADMET properties. As part of the analysis of the pharmacodynamics of the compounds, the ability of the drug to interact with a specific protein and other metabolic enzymes and transporters, as well as pharmacogenomics such as their effect on gene expression of various proteins and their possible adverse effects and side effects, were predicted using the Way2Drug PASS Online server [9]. In the program, the SMILES (Simplified Molecular Input Line Entry System) of the drugs analyzed were fed as the input for each drug. Ten interactions with the highest Pa value were considered. Pa (probability “to be active”) is an estimate of the probability of the drug belonging to the subclass of active compounds resembling the established set of activities in the server training set. Similarly, Pi is the probability “to be inactive” that is estimated by the server. The phytochemical properties, including lipophilicity, the extent of water solubility, pharmacokinetics, and drug-likeness, including topological polar surface area (TPSA), cytochrome inhibition, and gastrointestinal absorption ability, were assessed using the SwissADME online server program. Scores for each drug were tabulated.

## Results

The study was carried out to assess the predominant compounds that are present in the leaves of *M. koenigii* (Table 1) for their ability to interact with the immunodominant surface protein of *S. mutans* through bioinformatic analysis.

All 14 compounds were obtained from the PubChem database, converted into PDB format, and subjected to molecular docking with the glycosyltransferase protein of *S. mutans*. Among the 14 different compounds analyzed, the present study identified O-methyl murrayamine, koenigine, koenigicine, and murrayone to have inhibitory potential against the glycosyltransferase protein of *S. mutans* (Table 2).

**Table 2:** Molecular docking of O-methyl murrayamine, murrayone, koenigine, and koenigicine against S. mutans ASN, asparagine; E-value, binding free energy (kcal/mol); GLN, glutamine; GLU, glutamic acid; THR, threonine

S. no	Phytochemicals	Binding residues	E-value	No. of hydrogen bonds
1	O-methyl murrayamine	GLU-275	-268.64	1
2	Koenigine	THR-296	-275.92	1
3	Koenigicine	ASN-277	-285.21	1
4	Murrayone	GLN-279	-240.26	1

The binding affinity in the molecular docking experiment was evaluated in terms of the E-value and the number of hydrogen bonds formed between the compound and the ligand. Participation in electrostatic hydrogen bonding as a donor and acceptor, especially for noncovalent binders, is considered an important interaction apart from ionic interactions and van der Waals interactions. The binding energy (E-value) has been another important parameter to evaluate the binding affinity of any two molecules. The lower the E-value, the stronger the binding is. The highest negative energy value of the four compounds with hydrogen bonding indicates binding with the respective residues with strong binding affinity.

**Figure 1:**
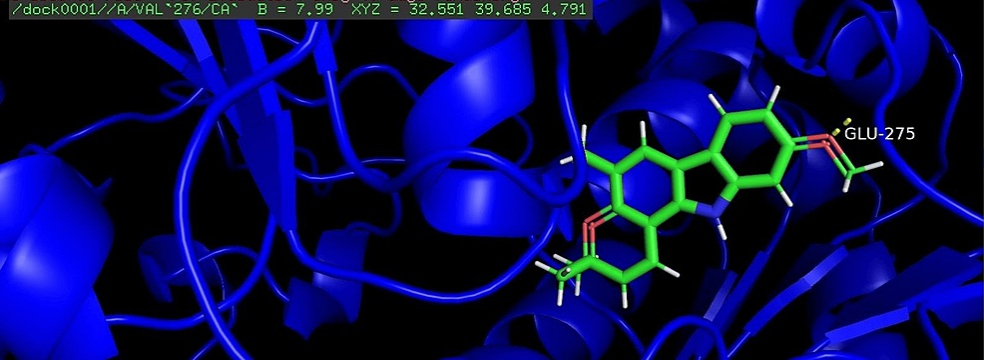
Molecular docking of O-methyl murrayamine with the glycosyltransferase protein of S. mutans The amino acid glutamic acid at position 275 (GLU-275) was identified as the interacting residue with the protein. GLU, glutamic acid

**Figure 2:**
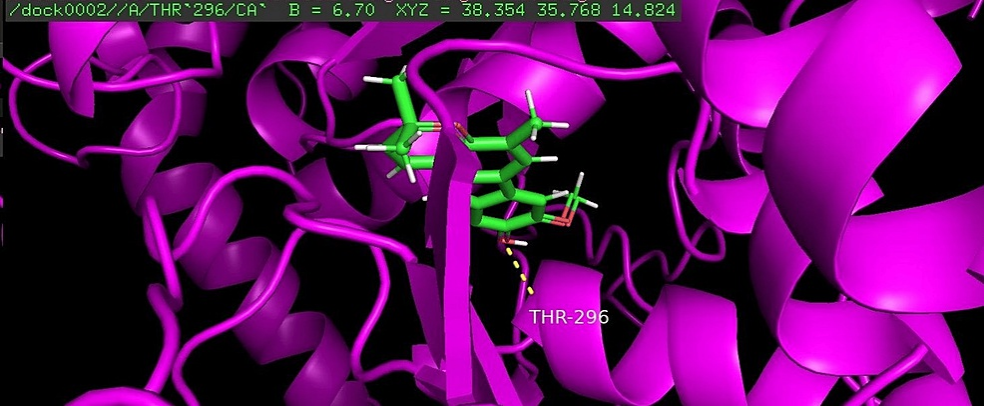
Molecular docking of koenigine with the glycosyltransferase protein of S. mutans The amino acid threonine at position 296 (THR-296) was identified as the interacting residue with the protein. THR, threonine

**Figure 3:**
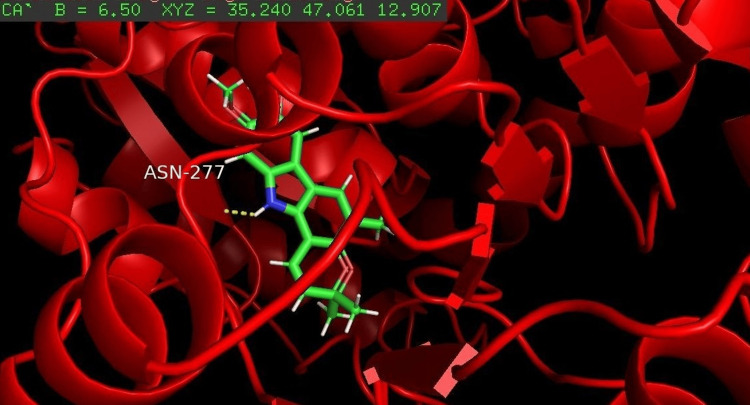
Molecular docking of koenigicine with the glycosyltransferase protein of S. mutans The amino acid asparagine at position 277 (ASN-277) was identified as the interacting residue with the protein. ASN, asparagine

**Figure 4:**
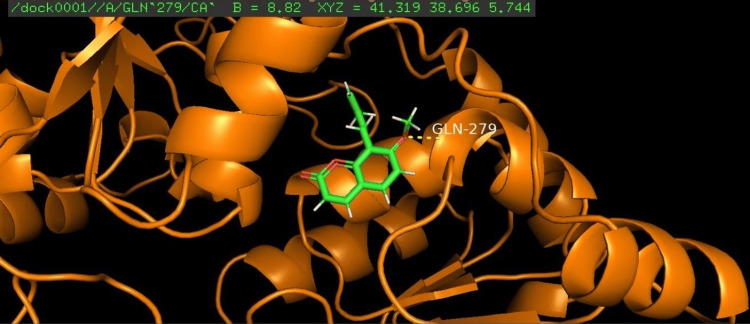
Molecular docking of murrayone with the glycosyltransferase protein of S. mutans The amino acid glutamine at position 279 (GLN-279) was identified as the interacting residue with the protein. GLN, glutamine

Among the four compounds analyzed for docking, koenigicine had the lowest E-score of -285.21, indicating a strong interaction with the receptor. The molecular docking of the four compounds was visualized, annotated, and highlighted using the PyMOL program (Figures 1-4).

Predicted metabolic activity (Pa and Pi) values were predicted, and the results were tabulated (Tables 3-6). Specifically, activity associated with glycosyltransferase enzyme function was not identified. O-methyl murrayamine was predicted to have CDP-glycerol glycerophosphotransferase inhibitor that functions on substitution of phosphate groups and apoptosis agonist activity. Koenigine and koenigicine were predicted to have hypoxia-inducible factor-1α (HIF1A) expression inhibitor activity that functions upon angiogenesis, and murrayone had aspulvinone dimethylallyltransferase inhibitor activity. The Pa and Pi values that indicate the probability of the activity as being active or inactive corresponded to the biological activity spectra of the compound tested. The estimation was based on the structure-activity relationship.

**Table 3:** Predicted metabolic activity of O-methyl murrayamine MYC, myelocytomatosis oncogene; Pa, probability of the compound to be active; Pi, probability of the compound to be inactive

Pa	Pi	Activity
0.814	0.027	CDP-glycerol glycerophosphotransferase inhibitor
0.778	0.009	Apoptosis agonist
0.751	0.002	MYC inhibitor
0.745	0.019	Antineoplastic

**Table 4:** Prediction of biological activity for koenigine CYP, cytochrome P450 family; HIF1A, hypoxia-inducible factor-1α; Pa, probability of the compound to be active; Pi, probability of the compound to be inactive

Pa	Pi	Activity
0.844	0.009	HIF1A expression inhibitor
0.714	0.024	Antineoplastic
0.713	0.055	CYP2C12 substrate

**Table 5:** Prediction of biological activity for koenigicine HIF1A, hypoxia-inducible factor-1α; Pa, probability of the compound to be active; Pi, probability of the compound to be inactive

Pa	Pi	Activity
0.798	0.012	HIF1A expression inhibitor
0.721	0.023	Antineoplastic
0.702	0.006	Cytochrome P450 stimulant

**Table 6:** Prediction of biological activity for murrayone CYP, cytochrome P450 family; Pa, probability of the compound to be active; Pi, probability of the compound to be inactive

Pa	Pi	Activity
0.919	0.005	Aspulvinone dimethylallyltransferase inhibitor
0.862	0.004	Cardiovascular analeptic
0.846	0.008	Gluconate 2-dehydrogenase (acceptor) inhibitor
0.839	0.003	CYP2A11 substrate
0.824	0.006	Apoptosis agonist
0.804	0.019	Chlordecone reductase inhibitor
0.812	0.033	CYP2C12 substrate
0.780	0.004	4-Nitrophenol 2-monooxygenase inhibitor
0.752	0.006	Spasmolytic, urinary
0.709	0.004	Free radical scavenger
0.711	0.024	Antineoplastic

The ADMET properties, or pharmacokinetic properties, were predicted for the four compounds (Table 7).

**Table 7:** ADMET pharmacokinetic properties of O-methyl murrayamine, koenigine, koenigicine, and murrayone ADMET, absorption, distribution, metabolism, excretion, and toxicity; BBB, blood-brain barrier; CYP, cytochrome P450 family; GI, gastrointestinal; Log Po/w, arithmetic mean of n-octanol-water partition coefficient values for lipophilicity; TPSA, topological polar surface area

Parameters	O-methyl murrayamine	Koenigine	Koenigicine	Murrayone
TPSA	42.09 Å²	54.48 Å²	43.43 Å²	56.51 Å²
Inhibitors of CYP1A2, CYP2C19, CYP2C9, CYP2D6, and CYP3A4	Yes	Yes	Yes	No
GI absorption	High	High	High	High
Consensus Log P_o/w_	5	3.78	4.12	2.56
Water solubility class	Poorly soluble	Moderately soluble	Poorly soluble	Moderately soluble
BBB permeant	No	Yes	Yes	Yes
Lipinski	Yes; zero violations	Yes; zero violations	Yes; zero violations	Yes; zero violations
Bioavailability score	0.55	0.55	0.55	0.55

A TPSA score of less than 140Å² is considered suitable as a candidate drug molecule. The TPSA score ranged from 42 to 56, indicating the usefulness of the drug molecules analyzed. Among the four compounds analyzed, murrayone had a high TPSA score. In addition, the TPSA score is considered an appropriate indicator for the two-dimensional quantitative structure-activity relationship that correlates the function with its chemical structure. The occurrence of drug-drug interactions is vital for a drug and its potential action. Cytochrome P450 isozymes such as CYP1A2, CYP2C19, CYP2C9, CYP2D6, and CYP3A4 play a key role in the metabolism of drugs through oxidation. Therefore, it is important to identify if the candidate drugs are inhibitory to these cytochromes. Among the four compounds tested, all three except murrayone inhibited the P450 isozymes. All four compounds had similar bioavailability scores. The bioavailability score ranges from 0 to 1. This property forecasts the permeability and bioavailability of the drug and is expressed in percentages. The scores of the four tested compounds were 0.55, indicating considerable bioavailability and a good pharmacokinetic property. The water solubility was poor for all four compounds tested. This indicates the need for the complexation of active molecules with water-soluble agents to enhance their solubility. Most of the drugs are actively transported or absorbed from the gastrointestinal (GI) tract. Those that have reduced water solubility are prepared in lipid-based formulations for improved bioavailability.

## Discussion

This study identified the phytochemicals of *M. koenigii*. O-methyl murrayamine, koenigine, koenigicine, and murrayone have inhibitory potential against the glycosyltransferase protein of *S. mutans*. In recent days, there has been an increase in demand for herbal products, so the phytochemicals of plants can be used in various ways for the preparation of different herbal products. The presence of phytochemicals, such as alkaloids, flavonoids, terpenoids, and saponins, indicates that the extract can be a potential antioxidant, antidiabetic, and anticancer agent. Alkaloids, flavonoids, and phenols, being aromatic in nature, help fight the generation of free radicals.

The various bioactive products, such as O-methyl murrayamine, koenigine, koenigicine, and murrayone, have potential antioxidant, anti-inflammatory, and antimicrobial activities. These findings had been bound similarly to other studies [8]. Phytochemicals have the ability to boost immunity, inhibit the formation of cancer cells, and shield DNA damage from causing cancer and other illnesses. Most antioxidant phytochemicals have been found to have anti-inflammatory effects. Inflammation is the result of tissue damage brought on by pathogens, injuries, chemicals, heat, or other factors. Damaged cells release chemicals including prostaglandins, bradykinin, and histamine. These substances induce fluid leakage into tissues from blood vessels, which results in swelling. The identified phytochemicals have been found to have potential antimicrobial activity. Antibacterial activity can be defined as a generic term for all active substances (active substances) that can inhibit bacterial growth, prevent the formation of microbial colonies, and kill microorganisms. These findings have been found to be similar to those of other studies [9]. The essential oil of *M. koenigii* reduced the activity of xanthine oxidase, which, in turn, reduced the production of superoxide radicals. However, in this study, the essential oil of *M. koenigii* was not studied.

Different phytocompounds derived from various plants have been similarly shown to be potent antibacterial and anti-inflammatory agents [10-13]. O-methyl murrayamine, koenigine, koenigicine, and murrayone showed the least binding energy with the glycosyltransferase of *S. mutans* protein. Among the four compounds analyzed, murrayone had a high TPSA score, which indicates acceptable absorption and permeation of the compound. All four compounds had similar bioavailability scores. Among the four compounds analyzed for docking, koenigicine had the lowest E-score, indicating a strong interaction with the receptor. All are inhibitors of CYP enzymes, except murrayone. GI absorption for all four compounds is high. O-methyl murrayamine and koenigicine are poorly water soluble, and koenigine and murrayone are moderately water soluble. All four compounds show zero violations of the Lipinski rule. As only molecular docking is done in this study, further in vitro studies can be done on *M. koenigii* to find properties like antioxidant, antidiabetic, anticancer, and anti-inflammatory and can infuse the compounds of this plant into herbal medicines [14-18].

Previous studies on this plant’s phytochemistry have shown that carbazole alkaloids are present. Some of these alkaloids have been shown to have antioxidant, antitumor, antibacterial, anti-inflammatory, anti-trypanoma, and anti-mosquito properties. Bioactive metabolites are abundant in *M. koenigii*. Nutraceuticals and plant-based medications have drawn interest from the scientific community because of their natural origins, cheap cost, and few side effects. Clinical research verified that *M. koenigii* is essential for the prevention of a number of disorders. It is now possible to completely or partially comprehend the molecular processes behind these actions in possible therapies because of the vast amount of knowledge that has been gathered in this area. Despite the fact that the plant has been shown to have medical value, scientists have given it little to no attention. Consequently, more research is necessary to clarify how each of the distinct phytochemicals contributes to the numerous pharmacological effects that it elicits.

The present study comprehensively analyzed the compounds of *M. koenigii* for their potential use in the human healthcare system. The study, however, suffered limitations, including the demonstration of scientific evidence in animal models and human clinical trials. The phytocompounds in general might potentially act synergistically with other compounds at different systemic concentrations, with limited predictability of the same through bioinformatic analysis. The role of other compounds, such as small peptides and transient proteins, which are believed to play a pivotal role, is not analyzed in this study.

## Conclusions

The present study concluded that the compounds O-methyl murrayamine, koenigine, koenigicine, and murrayone have inhibitory potential against the glycosyltransferase protein of *S. mutans*. The many roles that *M. koenigii* and its derivatives play in combinations of cell signaling pathways at numerous levels in a variety of illnesses account for the molecular processes underpinning these actions. O-methyl murrayamine and koenigicine were thus identified as potent and selective inhibitors of *S. mutans* and subsequent proliferation. According to the current research, *M. koenigii* may be a good source of bioactive chemicals that might help stop tooth decay. The possibility of creating antimicrobials from higher plants seems fruitful as it results in the creation of novel medications, which are necessary in the modern world. The rising incidence of tooth decay and other oral infections creates a predicament of bad oral hygiene, which, when left untreated, leads to major health issues, including cancer. The ever-expanding identification of investigational new drugs is thus warranted. As the present study shows, the identified compounds have inhibitory potential against *S. mutans*. Further, they can be used in the preparation of toothpastes and mouthwashes to prevent tooth decay.
